# Conditional Split Inteins: Adaptable Tools for Programming Protein Functions

**DOI:** 10.3390/ijms26020586

**Published:** 2025-01-11

**Authors:** Callum Shepherd, Makeba Lawson-Williams, Alexandria Holland, Adebayo J. Bello, Darren W. Sexton, Femi J. Olorunniji

**Affiliations:** School of Pharmacy & Biomolecular Sciences, Faculty of Health, Innovation, Technology and Science, Liverpool John Moores University, James Parsons Building, Byrom Street, Liverpool L3 3AF, UK

**Keywords:** split inteins, conditionality, protein *trans*-splicing

## Abstract

Split inteins are biological mechanisms for the operation of the spatiotemporal control of protein activities. They function through protein *trans*-splicing, in which their N- and C-terminal fragments are expressed contiguously with two protein halves. The subsequent self-excision upon recognition of the complimentary fragment yields a mature, complete, and functional protein. The conditional regulation of protein splicing through environmental factors or the attachment of regulatory modules can be used to determine when and where a protein will operate, providing potential novel approaches for engineering biology applications. This review will discuss current split intein applications and the mechanistic basis for novel species classification. These considerations can provide guidance in intein and extein engineering through activation strategies, in the design of spatial arrangements, and in taking advantage of unique reaction environments. This can pave the way for the future implementation of novel split intein discoveries and the selection of appropriate intein species and aid in designing novel protein engineering strategies.

## 1. Introduction

Over the past century, biotechnology has become one of the most crucial fields at the forefront of cutting-edge science; tools harnessed from nature have become fundamental to our way of life. Whilst biotechnologies have been harnessed—albeit unwittingly—for millennia, the conscious extraction of biological properties from nature has exponentially increased since the early 20th century, achieving previously unimaginable opportunities in the application of scientific principles.

Among the range of biological molecules with applications in biotechnology, proteins and their vast range of properties and functions have been most extensively utilised, with uses as enzymes, hormones, transporters, and key components in a host of therapeutic products. Enzymes are key to several biocatalytic processes, being able to promote finely tuned reactions that are largely inaccessible to common chemical processes.

The spatiotemporal regulation of activities is an essential element in the application of proteins in biotechnology. Without control over the activity, there is no regulation of when and where an enzyme or protein will act, resulting in unpredictable activities. For example, enzyme-dependent drug delivery or the assembly of cellular structures could not be optimised without careful regulation of the activities of the molecular machines that promote these functions. In contrast, the unregulated random synthesis of recombinant proteins can result in the production of a protein toxic to the cell and can therefore hinder protein production through the promotion of cell death. Avoiding protein toxicity is a fundamental requirement for successful recombinant protein production and is achievable through strict control of transcription and translation. Gaining temporal control of protein expression can reduce the toxic effect on the cell and facilitate successful production.

Inteins are proteins encoded within a protein [1] and are analogous to introns in DNA; they comprise a non-operative section of the polymer chain with regard to the primary function of the encoded protein or gene [2]. Both are spliced to yield a mature protein or gene, respectively. In contrast to introns, which are spliced prior to translation, intein splicing is a post-translational modification process [3]. The polypeptide sequences adjacent to an intein are called exteins [1]. Inteins are translated contiguously within a protein upon which self-splicing occurs, whereby the intein excises itself from the translated protein to generate a reconstructed scarless protein from its two extein fragments [1]. Post-translational intein splicing allows for a faster expression of protein activity in contrast to protein induction via transcriptional control. The rapid reconstitution of activities from non-functional precursor proteins offers a mechanism for the spatiotemporal regulation of activity. Control of intein splicing presents an opportunity to determine when and where a protein activity is initiated, making inteins a simple approach to achieving spatiotemporal control.

While inteins are diverse in structure and mechanisms, they share two domains that are conserved across most species: the homing endonuclease domain and the Hedgehog/INTein (HINT) domain. The homing endonuclease domain is an invasive domain that inserts into a specific “homing” site on a genome [4]. These domains are thought to have invaded contiguous inteins, thereby fragmenting them and giving rise to split inteins [5]. The HINT domain is the catalytically active domain for inteins; protein trans-splicing (PTS) continues in the absence of the homing endonuclease domain but is diminished in absence of the HINT domain [6,7]. Thus, the targeting of the homing endonuclease domain in a contiguous intein conveys an increased chance of yielding a functional split intein [8].

Inteins were first discovered in the yeast genome, where they are embedded in the vacuolar ATP synthase catalytic subunit A (VMA) gene [9,10]. Since then, subsequent discoveries of classes of inteins have been reported, and they have been found to be distributed across different domains of life, including viruses and bacteriophages. Using gene sequencing and bioinformatics, the diverse distribution of inteins has been documented and can be found in InBase (Intein database) [11] and in other databases in the National Center for Biotechnology Information (NCBI) (www.ncbi.nlm.nih.gov/gene; Accessed on 28 December 2024).

Inteins function in a natural system as autonomous protein splicing devices, thereby forming a system for the conditional expression of activities. They catalyse this process by removing themselves from a precursor polypeptide via the cleavage of two peptide bonds. This process is followed by extein ligation to form a new peptide bond. This polypeptide rearrangement occurs at the post-translational level [12,13]. Homing endonuclease (HEN)^2^ domain [6] are often present in inteins, which are naturally occurring mobile genetic elements. These inteins containing homing endonucleases function by moving their coding sequences into homologous alleles at homing sites lacking an intein sequence, resulting in intein horizontal gene transfer and vertical transmission [14].

The distribution and evolution of inteins, as well as their biological functions, have been extensively reviewed [3,15,16]. In this review, our focus will be on the mechanistic understanding of how split inteins function and their application in biotechnology and protein engineering.

## 2. Split Inteins

**Figure 1:**
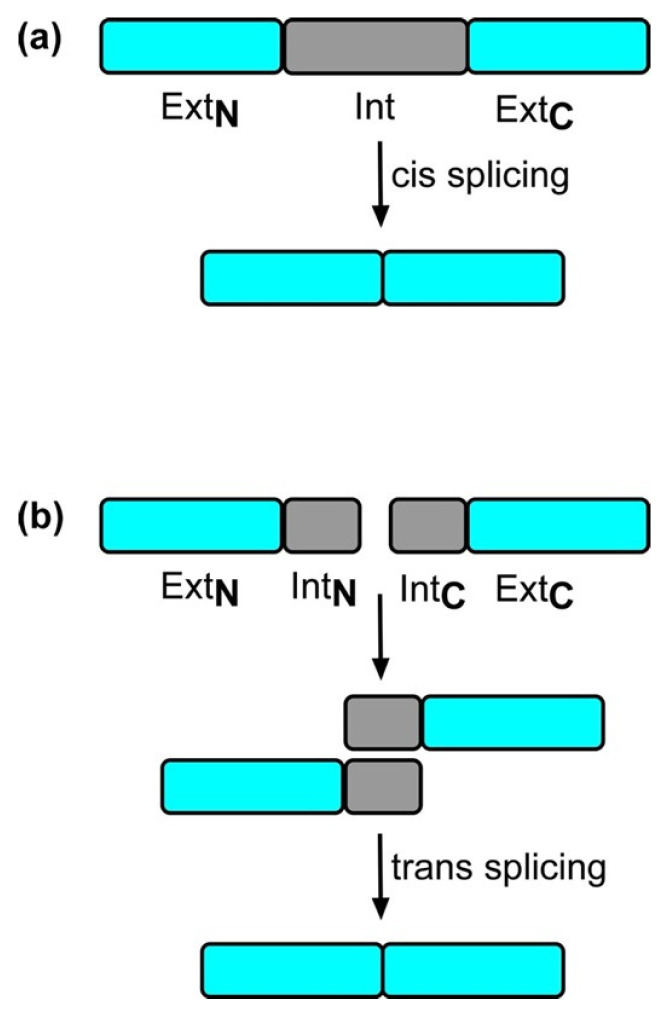
Protein trans-splicing by inteins. (**a**) The intein domain (gray) is sandwiched between the N-terminal extein (Ext**_N_**) and the C-terminal extein (Ext**_C_**) (both in cyan). Splicing in cis results in the reconstitution of a functional protein and the excision of the intein domain. (**b**) Non-covalent binding of the N-terminal intein (Int**_N_**) and the C-terminal intein (Int**_C_**), and a trans-splicing covalent reaction joins the N-terminal extein (Ext**_N_**) and C-terminal extein (Ext**_C_**) to reconstitute the active protein.

Contiguous inteins are situated between extein pairs. The extein pair, Ext_N_ (at the intein’s N-terminus) and Ext_C_ (at the C-terminus), are linked at each intein–extein interface by a peptide bond (Figure 1). Contiguous inteins undergo protein *cis*-splicing [17]. Split inteins operate similarly to contiguous inteins in that they are unrelated sequences that fragment a protein sequence and self-splice following translation; however, they are not expressed as one contiguous polypeptide [18]. The intein is split into two fragments (Int_N_ and Int_C_), which are linked to Ext_N_ and Ext_C_, respectively, by a peptide bond and are encoded by separate genes [18], therefore translating as two distinct proteins. Due to two individual intein fragments performing trans-splicing, a different mechanism is deployed; splicing cannot occur without association of the N- and C-terminal fragments; thus, split inteins undergo protein *trans*-splicing (PTS) [19].

Inteins are derived from a variety of bacteria, giving rise to a host of intein species. Whilst split inteins are found in nature, they can also be engineered through the splitting of a contiguous intein to yield two fragments [20]. There are also further possibilities of split intein construction through the variation of split sites on a contiguous intein [21] and through the alteration of a naturally occurring split intein split site [22]. From this variety of split intein species possibilities comes a variety of mechanisms dependent on different conserved functional residues, both on the intein and the extein.

## 3. Applications of Split Inteins

The range of split intein applications has only been partially discussed [8,23,24], but the literature highlights their diversity and importance to the biotechnology toolbox. The variety of systems in which split inteins can be used suggests that selecting the most appropriate split intein for utilisation could be a daunting prospect.

Inteins have been utilised for a plethora of applications in biotechnology due to the advantageous spatiotemporal control they offer over protein reconfiguration. They are widely used in recombinant protein ligation and labelling, and atypical split inteins are routinely used in protein labelling as the short fragment length permits their inclusion in a recombinant protein. Some examples are summarised below.

(1) The naturally split intein AceL-TerL is a rare, natural atypical split intein with an N-terminal fragment of 25 amino acids, offering unprecedented ligation of a fluorescent tag to a protein [25].

(2) Synthetic atypical split inteins *Ter*DnaE3 S11 and *Rma*DnaB S1 have been used for the internal FLAG-tag epitope labelling of maltose binding protein and thioredoxin for protein purification. This method can also be used for the fluorescent labelling and inclusion of chemical groups [26].

(3) Gp41-1 has been used to augment a split-Gal4 system in *Drosophila* for specific tissue labelling that is repressible by Gal80, offering novel temporal control [27].

(4) In a protein purification strategy, *Npu*DnaE_N_ is expressed in *E. coli*, and it captures its counterpart, *Npu*DnaE_C_, attached to a target protein, through strong native fragment association. Purification is subsequently achieved through a change in pH that promotes the dissociation of the captured fragment [28]. This approach demonstrates a process that efficiently yields an untagged native protein for characterisation or functional studies.

(5) The expression of a toxic protein as fragments has been used to reduce system toxicity, therefore highlighting the potential of split inteins as a means of obtaining spatiotemporal control [29]. It is worth noting that the expression of proteins as fragments can lead to misfolding and insolubility, likely through the exposure of hydrophobic regions, but this can be overcome by the expression of the two split protein fragments in the same cell or through refolding after the fragments have been purified [20].

(6) Adeno-associated vector (AAV) gene delivery systems are fundamental in protein therapeutics but are limited to 5 kb cargo packing [30]. A dual AAV delivery system has been designed where split inteins enable the reconstitution of a fragmented therapeutic protein in vivo, conveying a greater cargo size [31]. *Npu*DnaE, *Cfa*, and Gp41 split inteins have been utilised in AAV systems, with *Cfa* and Gp41-1 demonstrating 100% protein reconstitution and a rate that is two-fold better than *Npu*DnaE.

(7) A dual-signal AND logic reporter system has been developed using *Ssp*DnaE_C_ and *Npu*DnaE_N_ to produce a “digital-like signal” in prokaryotes using T7 RNA polymerase [31]. Int_c_ is induced by an arabinose-inducible promoter independently of the Int_N_ induction by IPTG. The induction of Int_C_ and Int_N_ by arabinose and IPTG is required for the intein-mediated ligation of flanking T7 RNA polymerase fragments to permit the expression of a green fluorescent protein adjacent to a T7 promoter, conveying a dual signal reporter system. In another study, the *Ssp*DnaE_C_ and *Npu*DnaE_N_ pair were used to design a split serine integrase system [32], demonstrating the potential for the spatiotemporal regulation of genome editing applications.

(8) An interesting technique for the spatiotemporal control of protein delivery is the concept of protein cages. Split inteins have been utilised in various designs of generic structures for diverse applications [33]. IMPDH-1 and Gp41-1 were used independently to build dimeric protein cages: split inteins associate and ligate the pyramidal half-cage proteins, attached to N- and C-terminal intein fragments, to form a bipyramidal structure. The orthogonality of IMPDH-1 and Gp41-1 was manipulated to facilitate the stepwise construction of larger generic protein cages. This highlights the importance of orthogonal intein pairs for compatible use in a single system.

## 4. Mechanisms of Protein *Trans*-Splicing Reactions

**Figure 2:**
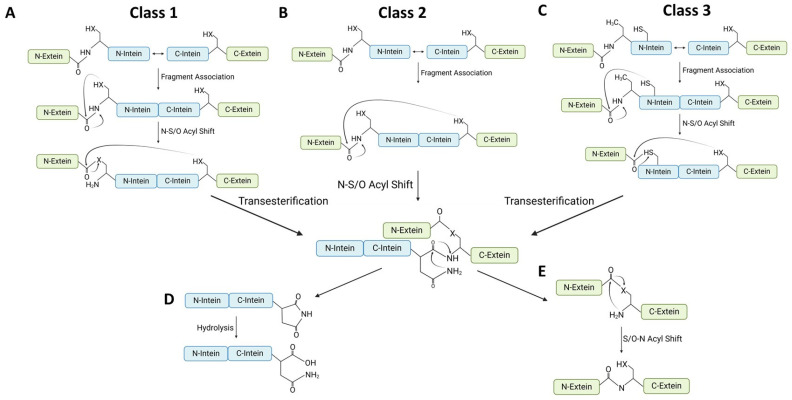
An overview of the Class 1–3 mechanisms of protein trans-splicing. (**A**) Class 1 mechanism: Recognition of C-/N-intein fragments through capture and collapse or threading for the reformation of the HINT fold, followed by a nucleophilic attack of catalytic Cys/Ser residue at a conserved 1 position, which causes a N-S/O acyl shift. Nucleophilic attack of branched intermediate at N-terminal splice junction by catalytic Cys/Ser/Thr residue at +1 position, which causes transesterification of the branched intermediate to the C-terminal Ext-Int interface. (**B**) Class 2 mechanism: Nucleophilic attack of N-terminal Int-Ext interface scissile peptide bond by upstream +1 catalytic Cys/Ser/Thr to form branched intermediate. (**C**) Class 3 mechanism: Nucleophilic attack of N-terminal Int-Ext interface scissile peptide bond by an internal Cys residue to create branched intermediate within the intein. Nucleophilic attack of branched intermediate by catalytic Cys/Ser/Thr residue at +1 position, which causes transesterification of the branched intermediate to the C-terminal Ext-Int interface. (**D**) Shared mechanism: Following the formation of branched intermediate at the C-terminal splice junction, all mechanisms follow the same pathway. Asparagine cyclisation of Intein C-terminal Asn residue, leading to succinimide ring formation and intein excision. (**E**) The S/O-N acyl shift of the branched intermediate reforms the peptide bond between the N- and C-terminal extein fragments for ligation.

The diverse nature of split intein species implies a variety of protein *trans*-splicing mechanisms. Naturally split inteins utilise different variations of a basic splicing mechanism depending on their native catalytic residues. In a similar way, synthetically split inteins mediate trans-splicing following a similar mechanism. Although the same general splicing principle is followed, the variety of species utilise different amino acid residues at the catalytic sites [34]. Although these residues tend to be functionally similar, they follow different thermodynamic pathways, hence the variations in the post trans-splicing mechanism [35]. To put split inteins to use for optimised specialised systems, it is necessary to describe the different species and the nuances of the mechanisms through which they work. Such background knowledge of the intricate and varied mechanisms would allow for the selection of the right split inteins to use for each unique application (see also Figure 2).

### 4.1. Canonical Split Inteins

To our knowledge, there is no comprehensive review that classifies the different species in terms of the rates of protein trans-splicing reactions or guides the engineering of a system dependent on split intein-mediated control. Due to the wide diversity of split inteins (>100 species) [36], this review will not cover every single species but will instead focus on the functionally significant ones. Our aim is to provide a classification of split intein species that serves as a guide for selecting the right ones for compatible and optimised applications. The classification described here has the potential to guide future discoveries of novel split intein species based on their characteristics and key catalytic residues and splice sites. The current classification of inteins [37,38] focuses solely on the protein trans-splicing mechanism. The classification described here adds another level that focusses on the splicing rate and the key catalytic steps involved in the transesterification reactions. This could guide future discoveries of novel split intein species based on their reaction rates, key catalytic residues, and splice sites.

Once expressed, naturally occurring split intein fragments will recognise their complementary counterpart and associate through a “capture and collapse mechanism” [39]. Int_N_ is expressed as a partially folded polypeptide with a disordered region, and Int_c_ exists as a totally disordered sequence. In the capture element of the mechanism, electrostatic interactions between extended, disordered anionic regions of Int_N_ and cationic Int_C_ initiate recognition of the respective fragments. In the collapse stage, fragment association induces a conformational change in the intein, leading to the compaction and stabilisation of the disordered intein regions. In the collapse element of the intein recognition mechanism, the pre-folded N-intein region subsides into the canonical intein fold. Association of the intein fragments to form the canonical intein fold is followed by the chemical steps of the spicing mechanism. Features of the three classes of split inteins are summarized in Table 1.

**Class 1 inteins** are the largest group and they follow the canonical protein trans-splicing mechanism (Figure 2A) [37]. Splicing is initiated by an N-S or N-O acyl shift (a reversible biochemical reaction involving the transfer of an acyl group from nitrogen to sulphur, or to oxygen during protein synthesis) through a nucleophilic attack of the scissile peptide bond at the Int_N_-Ext_N_ interface (using a conserved cysteine or serine at the catalytic position 1) to form a thioester or oxoester, respectively, resulting in a branched ester intermediate at an elevated thermodynamic state. Due to the thermodynamic nature of this elevated state, further structural changes are initiated. The first residue downstream of the intein (a conserved Cys, Ser, or Thr at catalytic position +1 on Ext_C_) initiates a nucleophilic attack on the thioester or oxoester from step 1, resulting in transesterification of ext_N_ onto the first downstream residue of the complimentary Ext_C_. Subsequent cyclisation of the terminal Int_C_ Asn results in the formation of an intein C-terminal succinimide ring (a ring formation occurring when nitrogen on peptide reacts with carbonyl carbon on the aspartyl and asparaginyl side chain, resulting in -AsnGly- motif rearrangement), followed by cleavage of the scissile peptide bond at the downstream Int_C_-Ext_C_ interface (Figure 2D). Following intein excision, the extein fragments are bound through a thioester or oxoester bond in the side chain of the downstream Ext_C_ +1 residue. Binding through this side chain is thermodynamically unfavourable, leading to a rearrangement to form a stable peptide bond through an intramolecular attack of the amino group. This results in a reconstructed and complete exteinm with no introduction of non-native amino residues (Figure 2E). Examples of Class 1 split inteins include gp41-1, with Cys1 and Ser+1 catalytic residues, [40] and *Neq*Pol, with Ser1 and Thr+1 catalytic residues [41].

**Class 2 inteins** utilise a protein *trans*-splicing pathway analogous to that used by Class 1 inteins (Figure 2B). Class 2 inteins use similar conserved residues that facilitate transesterification, Asn cyclisation, succinimide ring formation, and an ultimate intramolecular attack of the oxoester/thioester bond by the amino group (Figure 2D,E). In contrast to Class 1 reactions, protein trans-splicing reactions via Class 2 inteins (e.g., DnaB) do not involve an acyl shift at the N-terminal splice junction [38]. Transesterification occurs solely at the downstream Int_C_-Ext_C_ interface via an N-terminal nucleophile to form a thioester/oxoester branched intermediate.

**The Class 3 split intein** trans-splicing mechanism follows the general principle discussed above (Class 2 inteins), using a similar thermodynamic route (Figure 2C) and utilising the same key residues and the same chemical rearrangements. Like Class 2 inteins, Class 3 inteins do not involve rearrangements at the upstream Int_N_-Ext_N_ interface. However, unlike Class 2, an internal Cys residue side chain forms a thioester bond with ext_N_ to configure a branched intermediate before transesterification with the downstream splice junction and the canonical splicing events (Figure 2D,E) [38]. AceL-NrdHf is a typical example of a Class 3 split intein. It is a canonical intein in terms of fragment length but features an internal Cys in the C2 motif for branched intermediate formation [42].

The inteins in each class all share the requirement for a Cys or Ser residue at position 1 and a Cys, Ser, or Thr at position +1. Transesterification from these residues is thermodynamically favourable as branched intermediates at 1 and +1 positions become more stable following transesterification or maintain the same level of stability. There are evident preferences amongst inteins for the catalytic residues at 1 and +1 sites: Cys is the most common residue at position 1, with 36% paired with Cys+1, 29% paired with Ser+1, and 12% paired with Thr+1; and Ser is far more atypical, with Ser1/Ser+1 and Ser1/Thr+1 both found in 5% of protein trans-splicing reactions [34]. The data collected by Qi et al. [34] demonstrate an intein preference for the even thermodynamics of thioester to thioester or the downhill thermodynamics of thioester to oxoester of Cys1 over the oxoester chemistry used by Ser1. The Cys sulfhydryl group constitutes a superior leaving group in relation to the hydroxyl groups of serine and threonine, thus improving thioester lability in comparison to oxygen esters [37].

An uncommon protein trans-splicing residue combination reported by Bachmann and Mootz [35] is Ser1/Cys+1 in GOS-TerL. While this intein follows the canonical Class 1 splicing mechanism, it utilises a rare thermodynamic pathway. An oxoester bond is formed at the N-terminal splice junction, mediated by a serine nucleophilic attack of the scissile peptide bond, to form the upstream branched intermediate. Transesterification is subsequently facilitated by Cys+1 at the downstream splice junction to form a thioester branched intermediate before the canonical splicing events. It was reasoned that the shift from the unstable oxoester to thioester is supported by Ser87 distortion of the upstream Int_C_-Ext_C_ peptide bond to convey thermodynamic stability in favour of thioester formation through transesterification, hence overcoming the uphill thermodynamic nature of the shift. Furthermore, GOS-TerL is compatible with Cys1 residue, as would be expected based on other protein trans-splicing mechanisms since the process follows a thioester to thioester branched intermediate and a level energetic transesterification.

Whilst many canonical inteins are capable of sequence substitutions, it is worth noting that the alteration of conserved residues may alter the splicing efficiency or completely block the splicing activity. Whilst GOS-TerL uses a Ser1/Cys+1 combination, the same combination renders its closest homologue, Ace-TerL, inactive [35]. This highlights the importance of matching extein sequences compatible with the intein requirements for trans-splicing, both at splice junctions and at other parts of the protein sequence.

### 4.2. Atypical Split Inteins

Inteins with non-native split sites follow analogous trans-splicing mechanisms. However, due to non-canonical split sites, association between the split inteins occurs differently. Atypical inteins consist of one larger fragment and one small fragment, making them easier to include in a recombinant protein sequence with required extein sequences [21]. The larger intein fragment may exist as a near-complete intein with a cavity that must be filled by the smaller complementary cavity in order to assemble a functional trans-splicing complex [21]. Synthetic splitting of highly active contiguous mini-inteins at different positions can yield functional atypical split inteins for PTS applications [21]. These were made by separating contiguous mini inteins at varying points in the sequence, i.e., S1 split sites proximal to int-N terminus and S11 proximal to the int-C terminus. It was found that 50% of synthetic atypical inteins retain splicing capability. The authors reasoned that non-functional S1 and S11 splits may not be able to form a functional trans-splicing complex due to the occlusion or hinderance of short fragment docking in the larger fragment cavity.

Atypical inteins use canonical splicing mechanisms, but due to structural asymmetry, intein fragment pairs require a variant fragment association step for assembly of the canonical HINT domain [43]. Atypical inteins necessitate Ext_N_ threading through the Int_C_ during assembly. Stevens et al. [43] studied the synthetic intein consensus sequence via the Ace-TerL intein and found that the atypical intein fragments bind through clustered hydrophobic residues, followed by Glu2 Ext_N_ interaction with Ser75 and His78 on Int_C_, (rather than Glu+2 on Ext_C_). This implies the threading of Ext_N_ through Int_C_ to overcome the topological obstacle imposed by the requirement of an unusual HINT assembly for successful protein trans-splicing.

Intein pairs may demand explicit extein canonicity, such as Cat [43], due to their unique process, while others may not withstand non-canonical splitting as the trans-splicing complex may not be able to reconstitute the full intein. Thus, it is necessary to consider the topological requirements of an atypical split intein if it is to be deployed in a system. Atypical intein pairs will not follow the canonical association mechanism which brings about topological challenges. To overcome these factors, it is important to consider the mechanism of association required for the intein species, as well as the mechanism of trans-splicing to yield a functional intein.

## 5. Design of Conditional Protein *Trans*-Splicing Systems

**Figure 3:**
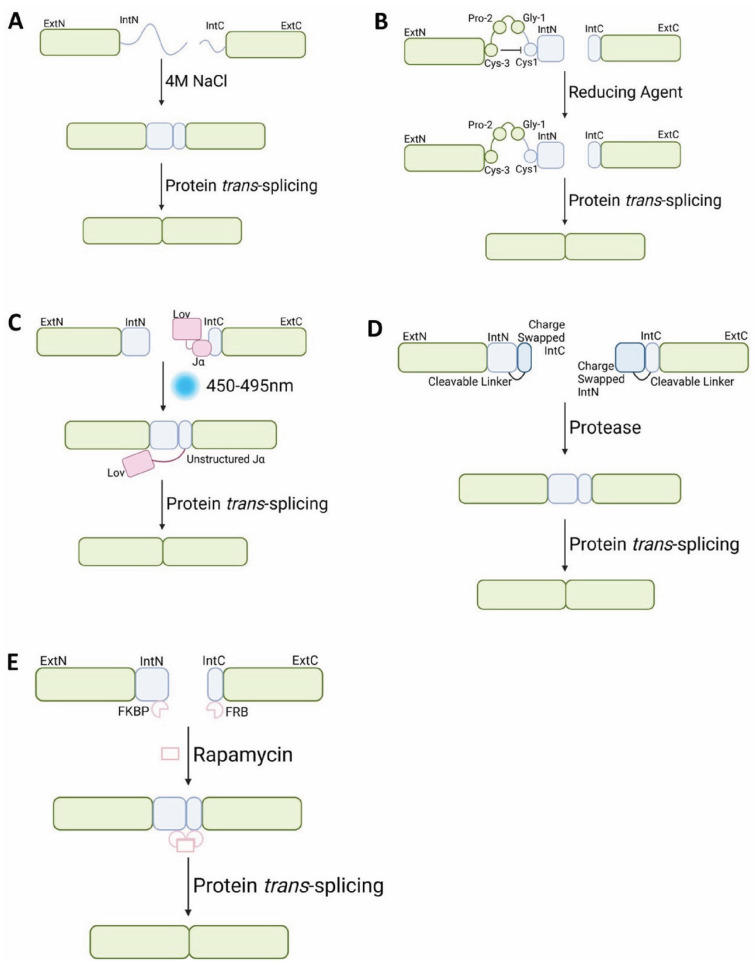
Selected examples of mechanisms for conditional split intein-mediated protein *trans*-splicing: (**A**) salt-induced protein *trans*-splicing [44,45]; (**B**) redox-induced protein *trans*-splicing [46]; (**C**) photocaging-induced protein *trans*-splicing [52]; (**D**) protease-induced protein *trans*-splicing [47,54]; (**E**) chemo-induced protein *trans*-splicing [57,59].

Protein *trans*-splicing can be developed into systems for reconstituting protein functions in biotechnology. One approach to achieving this is through conditional protein trans-splicing, which allows for the more precise timing of intein ligation to achieve tighter control on the expression of protein activity. Several strategies have been designed to control intein trans-splicing through conditional fragment association. This section aims to critically review the reaction environment conditions that may be exploited so that inteins may be readily integrated into a system. The inteins and the systems discussed below by no means comprise an exhaustive list but represent a selection of examples for the key principles discussed (see also Figure 3).

### 5.1. Salt-Induced Protein Trans-Splicing

Inteins derived from extremophiles possess characteristics that allow them to work under special conditions, which can be used as the conditional factor for activating trans-splicing. For example, halophilic inteins, such as MCM2 from the halophile *Halorhabdus utahensis*, are inactive under low-saline conditions, but trans-splicing is induced upon exposure to salt [44]. MCM2 utilises a catalytic Ser+1 for Class 1 PTS to facilitate protein ligation and segmental isotopic labelling.

Inspired by such halophilic inteins, the *Npu*DnaE splicing reaction has been engineered for activation in 2 M NaCl, facilitating conditional segmental isotopic labelling [45]. Due to *Npu*DnaE high residue promiscuity at splice junctions, 29 mutations were introduced to confer greater salt tolerance on the intein; positive residues were replaced by negative residues of the same size (Asn to Asp, Gln to Glu, Lys/Arg to Asp/Glu); i.e., 7 hydrophobic residues (Val, Ile, Leu) were replaced by Thr, Leu100 was replaced by Ser, and surface Glu/Asp were replaced by Ser residues. This preserved intein topology whilst altering its chemical profile to become inducible at a salt concentration >1 M and up to 4 M, conditions under which the precursor proteins were precipitated. Although slower than native *Npu*DnaE (1.4 × 10^−1^ s^−1^) [7], salt-inducible *Npu*DnaE facilitated trans-splicing at 6.8 × 10^−2^ s^−1^, a rate comparable to that of native halophilic inteins [45].

### 5.2. Redox-Induced Protein Trans-Splicing

Protein trans-splicing can also be regulated through the redox engineering of the splice junction. Callahan et al. used a synthetic disulphide bridge between mutant *Ssp*DnaE Ext_N_ Cys3 and Int_N_ Cys1 to inhibit thioester formation at the scissile peptide bond, preventing consequent transesterification and therefore preventing splicing [46]. The Cys3 mutant conveys a CXXC motif at the N-terminal splice junction; Pro and Gly are selected to fill the motif based on a similar thioredoxin motif that creates a tight backbone loop and promotes the formation of a disulphide cross bridge. The addition of a reducing agent restores the thiol functional group of Cys1, initiating thioester formation, which allows the canonical Class 1 trans-splicing mechanism to continue. This only allows for the initiation of intein trans-splicing in the presence of a reducing agent. This approach was also demonstrated with *Pab*MoA [46], suggesting that the introduction of the CXXC motif at the N-terminal splicing interface of an intein dependent on a catalytic Cys1 residue can convey conditionality by means of a redox switch.

### 5.3. Photocaging-Induced Protein Trans-Splicing

Photocaging is a method for conditional trans-splicing dependent on the light-triggered release of a protective photocage bound to the intein backbone [47,48]. Photolabile 6-nitroveratryl groups were attached to Gly19 and Gly31 of *Ssp*DnaE_C_ to decrease affinity for *Ssp*DnaE_N_ and interrupt splicing [47]. Photoprotection was alleviated by 20 min exposure to 365 nm UV light, which facilitated the full reconstitution of wt-*Ssp*DnaE splicing activity. A similar photocaging-dependent regulation was achieved via the introduction of an *O*-acyl isomer interruption to the protein backbone at the Ser35 of *Ssp*DnaE_C_ near the intein active site [49]. The photolabile group was inserted to interrupt splicing without affecting fragment affinity [49]. A photocleavable group (*Nvoc*) and a protease cleavable (Factor Xa protease) recognition sequence were attached to the *O*-acyl isomer Ser35 as protective groups to confer intein conditionality and alleviate splicing inhibition. UV light of 325 nm and Factor Xa protease were able to liberate *Ssp*DnaE_C_ protective groups and restore trans-splicing functionality. In this system, UV exposure resulted in the complete restoration of activity, while the effect of protease-mediated activation was partial. In a similar approach, *Npu*DnaE has been engineered to undergo conditional trans-splicing through light-dependent induction using photocaged Cys residue to reconstitute mCherry red fluorescent protein and human Src tyrosine kinase [48].

An improved method of intein photoconditionality is photoactivation; it offers a stimulus that is more sensitive to the protein system, alongside precise spatiotemporal delivery. The photosensitive protein LOV2 from *Avena sativa* [50,51,52] was fused to *Npu*DnaE_C_ to act as a conditional trans-splicing switch [46]. The presence of LOV2 sterically inhibits *Npu*DnaE fragment association and therefore blocks splicing. LOV2 undergoes a large conformational change upon induction by blue light (450 nm). In the absence of light, LOV2 is bound to its C-terminal helical extension (Jα), which is unlocked by covalent attraction between Cys450 and its flavin chromophore in the presence of blue light. The resulting conformational change unlocks Jα [50,51,52]. Thus, in the presence of blue light, the conformational change of LOV2 unlocks *Npu*DnaE_C_ and allows for the initiation of protein trans-splicing events. This approach is demonstrated in a study in which LOV2 was used to mediate the *Npu*DnaE reconfiguration of yellow fluorescent protein, RhoA ATPase reassembly, caspase 7 reassembly, and GCaMP2 reassembly for Ca^2+^ imaging [53]. Whilst operational, the conditional *Npu*DnaE-mediated trans-splicing was far slower than native *Npu*DnaE PTS, with a response time in hours rather than minutes [53].

### 5.4. Protease-Induced Protein Trans-Splicing

Vila-Perelló et al. previously described a conditional protein trans-splicing approach using Factor Xa proteolysis. This approach, termed intein zymogens, has been further demonstrated when caged inteins were liberated upon proteolytic cleavage to enable splicing reactions [49]. *Npu*DnaE fragments were fused to part of their complementary component (*Npu*DnaE_C_ with *Npu*DnaE_N_ 51–102 and *Npu*DnaE_N_ with *Npu*DnaE_C_ 1–30x2, with charges swapped on Int_N_ and its cage), locking the intein in a binding intermediate, with a protease cleavage sequence between the native fragments and their cages. The caged intein fragments exhibited no affinity for one another after 5 days, but this was restored by caspase-3 cleavage, and they underwent trans-splicing with a half time of 15 min. While this is considerably longer than the native *Npu*DnaE half time of 50 s [7], this approach allows for spatiotemporal control of the trans-splicing reaction.

Furthermore, *Npu*DnaE intein cage sequences were used to design Gp41-1, Gp41-8, and NrdJ-1 cages based on similar acitivity and local extein dependence [54]. Similar to the examples given above, these intein species were entirely inactive until cage inhibition was lifted via proteolysis by caspase-3. These examples demonstrate the potential applications of intein zymogen-conditional protein trans-splicing control over inteins with an analogous splicing mechanism. It is anticipated that a general zymogen trans spicing control system could be developed for each class of inteins.

In a different study, *Npu*DnaE was converted to an intein zymogen activated by the hepatitus C virus NS3/4A protease, allowing gp41-1, gp41-8 and NrdJ-1 to be activated by tobacco etch virus protease, human rhinovirus 3C protease, and thrombin, respectively [54]. The independent nature of these four intein species [7] with individual conditional protease control confers a highly coordinated orthognal system dependent on intein trans-splicing, facilitating the use of multiple spatiotemporally controlled inteins within one system such that each may demonstrate a unique response. Gramespacher et al. also demonstrated that *Npu*DnaE can be engineered to produce a pathogenic response [54]. In this design, *Npu*DnaE was fused to Lys16 and SH3B (potent anti-*Staphylococcus aureus* activity) with a ScpA protease cleavage sequence (secreted by *S. aureus*) for cage release, making *Npu*DnaE activity sensitive to *S. aureus*. The intein ligates antibacterial agents only when triggered by *S. aureus*.

### 5.5. Temperature-Induced Protein Trans-Splicing

Inteins can be made sensitive to temperature to confer conditionality to the system. *Sce*VMA-mediated Gal80 proteins were grown at 18 °C and 30 °C to induce a temperature-sensitive phenotype [55]. Temperature0sensitive inteins were restricted at 18 °C but active at 30 °C in *Drosophila*, conferring an intein with trans-splicing conditional to the temperature of the system.

### 5.6. Chemo-Induced Protein Trans-Splicing

*Sce*VMA has been used to pioneer an important intein conditionality system activated by rapamycin [56]. The intein exhibits an extremely slow trans-splicing reaction and therefore requires auxiliary dimerisation via a small molecule: rapamycin [20]. The dimerisation domains FKBP and FRB [57] are attached to the intein fragments and are dimerised upon rapamycin induction, which, in turn, brings *Sce*VMA into close proximity to induce trans-splicing [58,59]. This approach has also been used inversely to slow down the splicing reaction. *Npu*DnaE has an extremely high rate of protein trans-splicing. The approach designed for *Sce*VMA described above was applied to impose conditionality on the *Npu*DnaE splicing reaction. *Npu*DnaE fragments are caged with binding regions from their complementary fragment, and then FKBP is attached to the C-terminus of caged *Npu*DnaE_N_ and FRB to the N-terminus of *Npu*DnaE_C_. This design requires that FKBP and FRB must first dimerise through rapamycin induction to uncage the inteins, which allows for the subsequent *trans*-splicing activity [60,61]. The conditionality mechanisms for the discussed *trans*-splicing are highlighted in Table 2.

## 6. Choosing a Split Intein Pair

The first step in designing an optimally functional intein-dependent system is the selection of a suitable optimal intein pair. A key factor is the target application of the system, and there are a variety of intein systems available to choose from. Intein pairs can be classified based on their reactivity and affinity. It is also important to consider the extein dependency of an intein pair. Often, it may be necessary to engineer the intein/extein sequences to allow for protein trans-splicing without compromising the functionality of the reconstituted protein. Inteins promiscuous to their flanking extein residues are considered highly desirable for protein trans-splicing since they can be deployed to different applications without needing to change extein sequences.

### 6.1. Highly Active Split Inteins

Inteins that exhibit high affinity rapidly associate with their complementary fragment. This feature often translates to rapid PTS reactions. There are several highly active intein species from a range of bacteria which have all been successfully deployed in a variety of systems [23,24].

Due to their rapid trans-splicing reactions, conditionality can be imposed on high-speed intein species to control the spatiotemporal delivery of the extein load using the approaches discussed in the previous section.

Highly active protein trans-splicing systems have potential applications that can be used if conditionality regulatory modules are built in to achieve spatiotemporal control. Without such controls, high-speed inteins will splice as soon as the fragments are in proximity, which could limit the value of such systems.

The most-used high-speed intein of choice is *Npu*DnaE, from the *Nostoc punctiforme* DnaE gene. *Npu*DnaE is unrivalled in its trans-splicing rate, its promiscuity, and its efficiency. *Npu*DnaE has been found to consistently conduct splicing at a similar rate in different systems: 1.2 (±0.2) × 10^−2^ s^−1^ in vitro [62] and 1.4 (±0.3) × 10^−2^ s^−1^ in vivo [7]. The splicing reaction rate of *Npu*DnaE has set the benchmark for high-speed trans-splicing since its discovery, and it is likely that this will remain as the benchmark for the classification criteria of high-speed inteins. For an intein to fit the high-speed category, it must meet or surpass the rate of *Npu*DnaE. In addition to its exceptional PTS rate, *Npu*DnaE displays excellent efficiency with 98% extein ligation [63].

*Npu*DnaE is a member of a canonical family of DnaE inteins, e.g., *Ssp*DnaE, from *Synechocystis* sp. PCC6803 [18]. An intriguing characteristic of DnaE inteins is the cross reactivity of the complementary Int_C_ and Int_N_ fragments of a different species [18,32,64]. Whilst not all DnaE species meet the criteria for a high-speed intein, a synthetic intein has been derived from the conserved DnaE-effective region on the second shell adjacent to the active site. In this system, the 73-residue consensus sequence was termed *Cfa* [64]. *Cfa* demonstrates an elevated trans-splicing rate in comparison to its parent inteins with a rate of 5.0 × 10^−2^ s^−1^ at 37 °C (*Npu*DnaE 1.4 × 10^−2^ s ^−1^) [7], but it has an optimum rate at 80 °C of 1.4 × 10^−1^ s^−1^, an improved rate at higher temperatures [65]. Furthermore, *Cfa* retains high trans-splicing activity in the presence of chaotropic agents, with reported activities of 1.1 × 10^−1^ s^−1^ in 8 M urea and 1.0 × 10^−1^ s^−1^ 3 M GuHCl, which are significant improvements on the tolerance of *Npu*DnaE to these chaotropic agents [65].

More recently, a host of intein species were identified from metagenomic databases, including gp41-1, gp41-8, Nrdj1-1, and IMPDH-1 [7]. All these new species are reported to be highly active inteins and demonstrate superior protein trans-splicing rates to *Npu*DnaE (gp41-1, 1.4 × 10^−1^ s ^−1^; gp41-8, 4.5 × 10^−2^ s ^−1^; Nrdj1-1, 9.8 × 10^−2^ s ^−1^, and IMPDH-1, 8.7 × 10^−2^ s ^−1^ at 37 °C) [7]. Gp41-1, in particular, excels with—to the best of our knowledge,—the fastest splicing rate reported to date and exceptional efficiency [30], setting a new precedent as the best intein toolkit for rapid protein trans-splicing. As well as performing faster protein trans-splicing than *Npu*DnaE at 37 °C, gp41-1 outperformed *Npu*DnaE from 0 °C to 12 °C, and all novel species described in Carvajal-Vallejos et al. [7] outperformed *Npu*DnaE from 25 °C to 37 °C. Furthermore, all the new intein species demonstrate high-speed trans-splicing from 45 °C to 50 °C and were active up to 55 °C. Gp41-1 and IMPDH-1 were active in trans-splicing at 60 °C [7]. Whilst gp41-1 outperformed *Npu*DnaE in trans-splicing activity, it was more susceptible to chaotropic inhibition, with a rate of 1 × 10^−3^ s^−1^ trans-splicing in 4 M urea [7], whereas *Npu*DnaE retained high-speed trans-splicing at this concentration [56]. Gp41-1, like *Npu*DnaE, was severely inhibited by 6 M urea and entirely inhibited by >8 M urea [7], an indication that *Cfa* is preferred when PTS reactions are to be carried out in chaotropic environment [64].

Interestingly, unlike DnaE inteins, gp41-1, gp41-8, Nrdj-1, and IMPDH-1 all demonstrate orthogonality; i.e., they each function entirely independently of one another, recognising the respective complementary component in the presence of other intein components [7]. Together, these four highly active split inteins provide an orthogonal set available in the toolkit.

An attempt to design orthogonal inteins using gp41-1 charge mapping onto mini-inteins was carried out by Beyer et al. (2020) [65]. The charge map of gp41-1 was grafted onto a *Npu*DnaB contiguous mini intein to form CI-*Npu*DnaB_C_/_N_ (25% splicing), and further residual mutations yielded Oth-*Npu*DnaB_C_/_N_ (5% splicing). The construct CI-*Npu*DnaB_N_ showed no trans-splicing activity when partnered with Oth-*Npu*DnaB_C_ but facilitated 100% activity when paired with *Npu*DnaB_C_ [65]. This confirms that gp41-1 charge grafting onto *Npu*DnaB can be successfully engineered to orthogonal C-terminal fragments. However, Oth-*Npu*DnaB_C_ requires a complementary fragment for the pairing to work. Oth-*Npu*DnaB_C_ demonstrates 5% trans-splicing with its Oth-*Npu*DnaB_N_ counterpart and 23% activity with *Npu*DnaB_c_. Whilst Oth-*Npu*DnaB_C_ and *Npu*DnaE_C_ are orthogonal to CI-*Npu*DnaB_N_, the other N-terminal fragment in the mixture may not be. *Npu*DnaB_N_ demonstrated 77% activity with *Npu*DnaB_C_, and Oth-*Npu*DnaB_N_ was not tested with *Npu*DnaB_C_. Therefore, these inteins cannot be considered as fully orthogonal intein pairs.

An atypical, fast-splicing split intein has been designed from the consensus sequences of GOS-TerL and Ace-TerL—consensuses atypical-TerL (Cat-TerL) [43]. This atypical intein is the inverse of typical inteins and features a short, disordered, 30-amino-acids residue Int_N_ that folds onto the disordered region of a partially folded larger 104-residue Int_C_. This forms a catalytically active HINT fold through the canonical capture and collapse mechanism to catalyse trans-splicing at a fast rate of 1.6 × 10^−2^. This intein is unique in that it is a high-speed intein with a short Int_N_. Furthermore, it can tolerate unfavourable conditions (high temperature and high urea concentration) better than its parent inteins.

### 6.2. Low-Affinity Split Inteins

High-speed inteins are determined by their splicing rate, which is in direct correlation to the affinity of their fragments. It follows that inteins with a lower affinity will demonstrate a slower protein trans-splicing rate phenotype. The rationale for introducing conditional trans-splicing to these inteins is not just to restrict spatiotemporal activity. Another key objective is to increase the rate of splicing reactions by promoting fragment association between low-affinity intein parts. This is often achieved by using cages to bring components together to increase reaction rates and to gain spatiotemporal control of splicing activity.

As discussed earlier, the high-speed intein *Npu*DnaE is currently unique amongst the DnaE intein family for its PTS high reaction rate, making it one of the best-characterised inteins. *Ssp*DnaE is another well-characterised intein from the DnaE family. It facilitates PTS at a rate of 6.6 × 10^−5^ s^−1^ [66] and is therefore far slower than its cousin, *Npu*DnaE. The intein depends on Cys1 and Cys+1 catalytic residues to mediate a Class 1 mechanism of PTS [18].

*Ter*DnaE3 is another member of the DnaE intein family from cyanobacteria and has been artificially split at positions S1 and S11, with splicing rates of 3.8 × 10^−4^ s^−1^ and 2.2 × 10^−4^ s^−1^, respectively [21]. The short fragments that are produced by artificially splitting an intein at S1 and S11 are advantageous for site-specific labelling as the short fragments are easy to incorporate into a target gene to facilitate the trans-splicing of a recombinant gene onto the target [67].

*Ssp*DnaB is another intein from *Synechocystis* sp. PCC6803. It is classified as a low-affinity intein and undergoes trans-splicing at rate of 7.14 × 10^−4^ s^−1^ [20]. *Ssp*DnaB has also been synthetically split at a range of different sites on loops between β-strands and into three fragments by splitting at S1 and S0. The synthetic *Ssp*DnaB splits demonstrate a PTS efficiency analogous to the native intein, and interestingly, the triple fragment intein is more efficient [68]. While some low-affinity inteins can be brought into close proximity via dimerisation domains to increase their reaction rates, *Ssp*DnaB does not benefit from rapamycin dimerisation induction and demonstrates no change in trans-splicing activity [20]. This could be explained by the higher thermostability of *Ssp*DnaB than *Sce*VMA, the model intein for rapamycin induction [20].

Other low-affinity intein species from *Synechocystis* sp. PCC6803 have been characterised. *Ssp*GyrB features a short Int_N_ and a long Int_C_ with a rate of 6.9 × 10^−5^ s^−1^ [67]. This intein has since been artificially split at S1 to form a short Int_N_ and a longer Int_C_ with an improved trans-splicing rate to 1.2 × 10^−4^ s^−1^ [69]. A drawback is that *Ssp*GyrB S1 is non-functional without native extein residues. *Ssp*DnaX is a synthetic intein split from a contiguous mini intein at various sites, with rates of 1.7 × 10^−4^ s^−1^ and of 1.9 × 10^−4^ s^−1^ for S1 and S11, respectively [21]. *Ssp*DnaX S1 is of particular interest to the intein toolbox. Like *Ter*DnaE-3, *Ssp*DnaB, and *Ssp*GyrB, it features a short Int_N_ fragment, which allows for the easy splicing of synthetic proteins onto a protein of interest [22]. Furthermore, *Ssp*DnaX S1 shows activities at temperatures ranging from 1 °C to 37 °C [22]. It is likely that mutagenesis of this species could help identify temperature-sensitive inteins, which afford conditional protein trans-splicing for site specific labelling.

Various species of low-affinity inteins have been used in a mixture to orthogonally attach fluorescent labels to proteins in a mixture [20,70]. This shows that low-affinity inteins can be used to build a toolbox of orthogonal splicing systems to achieve more intricately controlled systems.

*Sce*VMA is an important model of a low-affinity intein. It is synthetically split into two fragments that undergo PTS at an initial rate of 7.1 × 10^−4^ s^−1^. However, dimerisation with rapamycin induction of FKBP and FRB increases the splicing rate to 1.2 × 10^−3^ s^−1^ [20]. When induced by rapamycin, *Sce*VMA has a trans-splicing rate greater than most other low-affinity inteins, an indication that enhancement of trans-splicing rates can be achieved through dimerisation. *Sce*VMA is one of the aforementioned low-affinity inteins that can be used orthogonally, alongside *Ssp*DnaB or *Ssp*DnaE [20,71].

GOS-TerL is the first intein featuring Ser1/Cys+1 catalytic residues [35]. The thermodynamic requirements of this protein trans-splicing pathway may account for the slow reaction rate: 2.4 × 10^−3^ s^−1^ at 37 °C for GOS-TerL [35]. Interestingly, GOS-TerL exhibits an optimum rate of 4.6 × 10^−3^ s^−1^ at 30 °C [35]. It is a naturally occurring atypical intein with an Int_N_ of 37 amino acids that can be reduced to 27 and interchanged with the corresponding 25 amino acid fragment of the intein’s close homolog, AceL-TerL [35], an indication that it could exhibit high promiscuity. The combination of GOS-TerL promiscuity and its short N-terminal fragment could make the intein a good candidate for protein labelling. AceL-TerL, a homologue of GOS-TerL, is another low-affinity intein, with a short Int_N_, making it suitable for the modification of recombinant proteins [25,72]. Ace-TerL was isolated from an Antarctic saline lake, and as expected, it shows an optimum trans-splicing rate of 1.7 × 10^−3^ s^−1^ at 8 °C [25]. The intein is also functional at 25 °C and 37 °C, albeit at a lower rate. These observations suggest an opportunity for engineering the AceL-TerL intein species to obtain temperature conditionality and perhaps salt-inducible conditionality due to its native saline conditions.

## 7. Dependence of Protein *Trans*-Splicing Efficiency on Exteins

The nature of extein residues local to intein splice junctions are key factors in determining the success of all three classes of intein-mediated protein trans-splicing reactions. Without +1 catalytic residues, protein ligation is severely hindered [43]; therefore, all species of intein depend on their extein sequences. Certain alterations to native exteins can impede trans-splicing. Hence, it is essential to consider the innate dependence of an intein on its extein residues for ligation of non-native proteins. Conserved catalytic residues must be maintained to successfully engineer exogenous proteins. Despite this, techniques have been developed to overcome obstacles posed by the specific requirements of extein dependence and, in some cases, augment the trans-splicing reactions. When used in this context, the term “promiscuity” describes the attributes of an intein that functions with little to no extein canonicity.

Intein promiscuity is the gold standard strived for in the field as it makes inteins very easy to incorporate into a protein. As different intein species utilise different trans-splicing mechanisms, and each have unique characteristics, they each have a unique extein dependence and therefore a unique degree of promiscuity.

*Npu*DnaE is an excellent species for the demonstration of promiscuity. Protein ligation efficiency is maintained and even surpassed in non-native extein ligation reactions [73,74] due to its reactivity with non-native exteins at the splice junctions [63,75]. This attractive characteristic confers the requirement of only a few key residues, facilitating easy inclusion within a non-native flanking protein. *Npu*DnaE was found to require little Ext_N_ residue canonicity, with the only requirement being flexibility at the upstream splice junction with preference to Gly at -3 and -2 positions [74]. Ext_C_ displayed slightly more residual requirements but was still amenable to alterations, namely, a conserved Cys+1, relaxed specificity at +2 but a preference for Trp rather than native Phe, and a preference for native Asn+3 with some tolerance to Glu+3 and Tyr+3 [74]. The flexibility displayed by *Npu*DnaE regarding residual alterations at both splice junctions indicates the adaptability of flanking exteins and shows that not only can the intein retain its excellent function with non-native exteins; it can also demonstrate improved trans-splicing reactions. Whilst *Npu*DnaE favours certain residues at the +2 position, sequence changes away from the splice junction can be used to engineer the protein for more flexibility. Since the +2 residue interacts with the His125 loop of the active site, mutating Asp124 to Tyr can improve tolerance of a less bulky +2 amino acid such as Ala [75].

The engineered *Cfa* intein can also be engineered to show a high degree of promiscuity and therefore little extein dependence [76]. *Cfa* residues 122–124 can be mutated from Glu-Arg-Asp to Gly-Glu-Pro, allowing the native His125 to interact with a non-native Ala+2. This permits functional protein trans-splicing at a native rate with a non-canonical +2 residue and demonstrates an artificial induction of intein promiscuity.

Despite the success reported for the cases mentioned above, some inteins have not been engineered to support extein promiscuity. The highly active gp41-1 also demonstrates a poor degree of promiscuity in comparison to *Npu*DnaE and *Cfa*. Gp41-1 has been shown to be sensitive to extein alterations [65]. This could be due to the orientation of the conserved residues close to the splice junctions. The Gp41-1 N-terminal residue Cys1 side group is oriented away from the C-terminal Asn125, conferring an open conformation similar to other inteins [77]. Residual orientations such as this should be conserved when the intein is used along with heterologous exteins.

## 8. Designing the Spatial Arrangement of a Conditional *Trans*-Splicing System

The evident advantages offered to a system by conditional protein trans-splicing are only accessible if they are used in an integrated way that is compatible with other components of the system. The primary way to do this is through the selection of an appropriate split site that is favourable to the intein and to the protein. It is essential to retain the catalytic residues of the intein fragment to initiate and complete the trans-splicing reaction. Hence, there is a requirement that those residues are present in the construct. Whilst these residues may already reside within the protein sequence, in most cases, it may be necessary to introduce these residues for the insertion of a conditional protein trans-splicing system [32].

In deciding on a split site for a protein, it is necessary to consider whether changes to the selected split site where intein sequences will be inserted will affect the function of the reconstituted protein. Any changes to the protein sequence after the splicing reaction should not significantly affect the structure and, hence, the function of the protein. This is especially significant for modular proteins since domain interactions should not be affected by the splicing reaction.

Furthermore, if conditionality imposed changes in the reaction environment are to be included, their effects on the protein structure and function must also be considered. For example, a salt-inducible conditional system may alter protein folding through the variation of residue charges, which may render a protein incapacitated [78]. A temperature-dependent conditional system may also be unsuitable as temperatures required for the activation may denature or inactivate a protein. Similarly, conditional regulation, which depends on structural changes, may present further challenges. Caged inteins and photocaged and molecular inducible inteins may impose steric obstacles upon the protein system through interference at the active site.

These potential barriers to effective conditional protein trans-splicing can be circumvented by carefully considering the features of each intein and the corresponding extein. By selecting an appropriate conditionality system, a suitable split site, and a matching split intein pair, the technology can be used to achieve seamless reconstruction of the protein with fully functional activity.

Although there is an abundance of intein species available for use, navigating the intricacies of reaction environmental conditions and extein compatibility requires careful considerations. As previously demonstrated, every species has different characteristics and thus usually entail different conditions for their system.

Table 3 summarises the key features of the selected split inteins with emphasis on factors that may need to be taken into consideration when selecting which pairs to use in designing conditional protein trans-splicing systems. Table 4 provides some of the applications of split-inteins.

## 9. Conclusions and Prospects

Inteins utilise a conserved mechanism of protein trans-splicing without the need for accessory proteins or external input, making them amenable for use in engineering biology applications. The broad applications of inteins for biotechnology are evident, and they include bioconjugation, in vivo delivery, protein labelling, protein purification, and logic reporter systems (Table 2). Furthermore, there is a wide range of inteins with unique and subtle features at our disposal, which means they can be adapted to be included in most systems. Conditionality systems such as zymogen, photocages, and environmental regulators have been engineered to create sophisticated mechanisms of imposing spatiotemporal control that facilitate highly coordinated protein ligation.

As further work is undertaken to understand the reaction mechanism and create increased extein promiscuity, inteins will become more amenable to individual systems rather than dependent on native residues. Such a development will broaden the use to which intein systems can be applied. We predict that more orthogonal intein species from metagenomic studies, such as GOS-TerL and Ace-TerL, will be discovered, thereby facilitating the extended orthogonal protein trans-splicing of different proteins in the same system. This will be key for complex multi-labelling processes and metabolic constructs. Inteins already are an exciting technology for biotechnologists and will undoubtedly remain an important tool for future developments.

We predict that novel technologies for studying protein structure and function will enhance the usefulness of split inteins in the near future. For example, recent developments in the use of protein structure prediction and protein–protein interaction modelling tools, such as AlphaFold2 [87] and AlphaFold Multimer [88], will allow for a better understanding of how the addition of intein modules to the target extein could affect both the splicing efficiency and the functions of the spliced protein. This approach could streamline the design process and reduce the number of constructs to be tested experimentally. Similarly, AlphaFold3 can be used to enhance the specificity of small molecule ligands for conditional regulation of protein trans-splicing [89].

We also expect that the increasing use of machine learning in integrating the existing data on inteins’ sequence, structure, and mechanism with the sequence, structure, and functional requirements of exteins will help automate decisions on which split inteins to use for a desired application. To achieve this, detailed knowledge of the molecular structure and mechanism of the target extein protein will be needed. Hence, the application of split inteins in the programming of protein functions is likely to be more successful for proteins whose structures and functions are well characterised.

## Figures and Tables

**Table 1:** Summary of the characteristics of the reaction mechanisms of Class 1, 2, and 3 split inteins.

	Class 1 [37]	Class 2 [38]	Class 3 [38]
Fragment Association	Capture & Collapse	Capture & Collapse	Capture & Collapse
Intermediate formation (N-S/O acyl shift)	Int1 residue attack of peptide bond at N-terminal splice junction	Ext+1 residue attack of peptide bond at N-terminal splice junction	Internal intein residue attack of peptide bond at N-terminal splice junction
Transesterification	Ext+1 attack of intermediate	Intermediate forms directly at Ext+1	Ext+1 attack of intermediate
Intein Cleavage	Succinimide ring formation at intein C-terminal Asn, leaving branched Ext_N_-Ext_C_	Succinimide ring formation at intein C-terminal Asn, leaving branched Ext_N_-Ext_C_	Succinimide ring formation at intein C-terminal Asn, leaving branched Ext_N_-Ext_C_
Extein Linearisation	S/O-N Acyl Shift	S/O-N Acyl Shift	S/O-N Acyl Shift
Examples	Gp41-1 [40]	Contiguous MBe DnaB [41]	AceL-NrdHF [42]

**Table 2:** Systems for designing conditional protein *trans*-splicing systems.

Conditionality Mechanism	Accessory Elements	Sequence Editing	Environment Dependent	Induction Stimulus	Mechanism of Action
Salt induction [43,45]	No	Yes—Intein	Yes	Salt	Salt induces a conformational change of the intein that reconstructs PTS capabilities, diminished by low saline conditions.
Redox induction [46]	No	Yes—Extein	No	Reducing agent	The active Cys1 is sequestered by a novel Cys-3 on Ext_N_, which is liberated by a reducing agent to reconstitute PTS.
Photocaging [47,48,49]	No	Yes (Chemical)—Intein	No	365nm UV light	Addition of a chemical protective group to an intein fragment that reduces affinity for its counterpart. Photo-liberation of the protective group restores intein affinity and therefore PTS activity.
Optogenetic induction [53]	Yes	No	No	450nm Blue light	A light sensitive Lov2 domain attached to an intein fragment occludes PTS, which is restored following light-dependent reconfiguration of Lov2.
Protease induction [54]	Yes	No	No	Specific Protease	Intein fragments are sequestered by charge-altered, complimentary fragments, linked by a protease cleavage sequence. Addition of a specific protease cleaves the cage and restores PTS function to the intein pair.
Temperature induction [55]	No	Yes	Yes	Temperature	Random mutagenesis of the intein to yield an intein sensitive to temperature.
Chemo induction [12,56,60,61]	Yes	No	No	Rapamycin (dimerisation domain specific)	Dimerisation domains are attached to intein fragments to regulate their affinities. Dimerisation induction by an induction agent brings the intein fragments into close enough proximity for PTS to occur. This can be used to augment or repress PTS rates.

**Table 3:** A selection of typical and atypical intein species and their key reaction components.

Intein Species	Optimum Temp. (°C)	Temp. Range (°C)	Reaction Rate (*k* s^−1^)	PTS Rate Class	Int_N_ Size (No. of Residues)	Int_C_ Size (No. of Residues)	Position 1 Residue	Position +1 Residue	Mechanistic Class
Gp41-1 [7,39]	45	0–60	1.8 × 10^−1^	High	88	37	Cys1	Ser	1
Gp41-8 [7]	37	25–55	4.5 × 10^−2^	High	94	50	Cys1	Ser	1
Nrdj1-1 [7]	37	25–55	9.8 × 10^−2^	High	110	46	Cys1	Ser	1
IMPDH-1 [7]	37	25–60	8.7 × 10^−2^	High	106	42	Cys1	Ser	1
NpuDnaE ^†,‡^ [7,62]	37	0–65	1.4 × 10^−2^	High	102	36	Cys1	Cys	1
Cfa DnaE [64,79]	80	15–80	1.3 × 10^−1^	High	101	36	Cys1	Cys	1
Ssp DnaE [39,66]	*N.R.*	4–44	6.6 × 10^−5^	Low	123	36	Cys1	Cys	1
Ssp DnaB [20]	25	25–37	7.1 × 10^−4^	Low	106	48	Cys1	Ser	1
Ssp DnaB S1 [21,80]	25	25–37	1.7 × 10^−5^	Low	13	141	Cys1	Ser	1
Ssp GyrB [67,81]	19	19–37	6.9 × 10^−5^	Low	115	41	Cys1	Ser	1
Ssp GyrB S11 [20,67]	19	19–37	6.9 × 10^−5^	Low	150	6	Cys1	Ser	1
Ssp DnaX [20,22,81]	37	0–37	1.7 × 10^−4^	Low	94	39	Cys1	Cys	1
Ssp DnaX S1 [20,22,81]	37	0–37	1.7 × 10^−4^	Low	11	122	Cys1	Cys	1
Ssp DnaX S11 [20,22,81]	37	0–37	1.9 × 10^−4^	Low	127	6	Cys1	Cys	1
Mxe GyrA [82]	25	4–50	*N.R.*	*N.R.*	119	79	Cys1	Thr	1
Neq Pol [41]	80	50–100	*N.R.*	*N.R.*	98	30	Ser1	Thr	1
GOS-TerL ^‡^ [35]	30	8–37	4.6 × 10^−3^	Medium	37	115	Ser1	Cys	1
AceL-TerL ^†^ [25,43]	8	8–37	1.7 × 10^−3^	Medium	25	104	Cys1	Cys	1
Cat-TerL ^‡^ [43]	50	1–50	1.6 × 10^−2^	High	30	104	Cys1	Cys	1
Sce VMA [20,56]	25	25–37	9.4 × 10^−4^ *	Low	184	65	Cys1	Cys	1
MCM2 [44]	*N.R.*	*N.R.*	4.5 × 10^−4^	Low	117	41	Cys1	Ser	1
AceL-NrdHF [42]	37	8–42	3.1 × 10^−5^	Low	98	48	Cys124	Ser	3

* Reaction rate with FKBP/FRB heterodimerisation domains. ^†^ Native or mutant saline tolerance. ^‡^ Some degree of extein promiscuity. N.R., not reported.

**Table 4:** Examples of split-intein applications with potential for conditional regulation.

Application		Intein Species	Reference
Protein labelling	Fluorescent tag ligation onto protein N-terminus	Ace-TerL	[25]
	Flag-tag epitope internal labelling, fluorescent tags, chemical tags	*Ter*DnaE3 S11	[26]
		*Rma*DnaB S1	[26]
	Split Gal-4 *Drosophila* labelling	Gp41-1	[27]
	In vivo fluorescent labelling of neurogenerative stem cells	*Sce*VMA	[83]
Protein Purification	Purification of an untagged protein	*Npu*DnaE	[28]
	Purification of untagged recombinant therapeutic proteins	*Npu*DnaE	[84]
Recombinant Protein Production	Expression of a toxic/large protein as fragments to reduce cytotoxicity	Mxe GyrA	[29]
Protein Therapeutics	A dual-AAV system to deliver large cargo sizes	*Npu*DnaE *Cfa* Gp41-1	[30]
	Protein cage construction for delivery of therapeutic proteins	IMPDH-1 Gp41-1	[33]
	Reduced non-specific cytotoxicity in cancer treatment	*Npu*DnaE	[29]
	Novel bacteriocin detection	*Npu*DnaE	[85]
	SICLOPPS peptide library screening of tumour inhibitors	*Ssp*DnaE	[86]
Logic Systems	Split T7 RNA polymerase required for transcription of target protein	*Npu*DnaE_N_/*Ssp*DnaE_N_	[31]
	Split ϕC31 integrase for colour change reporter system	*Npu*DnaE_N_/*Ssp*DnaE_N_	[32]

## Data Availability

Not applicable.

## References

[1] Perler F.B., Davis E.O., Dean G.E., Gimble F.S., Jack W.E., Neff N., Noren C.J., Thorner J., Belfort M. (1994). Protein Splicing Elements: Inteins and Exteins—A Definition of Terms and Recommended Nomenclature. Nucleic Acids Res..

[2] Colston M.J., Davis E.O. (1994). The Ins and Outs of Protein Splicing Elements. Mol. Microbiol..

[3] Wang H., Wang L., Zhong B., Dai Z. (2022). Protein Splicing of Inteins: A Powerful Tool in Synthetic Biology. Front. Bioeng. Biotechnol..

[4] Edgell D.R. (2009). Selfish DNA: Homing Endonucleases Find a Home. Curr. Biol..

[5] Dassa B., London N., Stoddard B.L., Schueler-Furman O., Pietrokovski S. (2009). Fractured Genes: A Novel Genomic Arrangement Involving New Split Inteins and a New Homing Endonuclease Family. Nucleic Acids Res..

[6] Derbyshire V., Wood D.W., Wu W., Dansereau J.T., Dalgaard J.Z., Belfort M. (1997). Genetic Definition of a Protein-Splicing Domain: Functional Mini-Inteins Support Structure Predictions and a Model for Intein Evolution. Proc. Natl. Acad. Sci. USA.

[7] Carvajal-Vallejos P., Pallisse R., Mootz H.D., Schmidt S.R. (2012). Unprecedented Rates and Efficiencies Revealed for New Natural Split Inteins from Metagenomic Sources. J. Biol. Chem..

[8] Li Y. (2015). Split-Inteins and Their Bioapplications. Biotechnol. Lett..

[9] Kane P.M., Yamashiro C.T., Wolczyk D.F., Neff N., Goebl M., Stevens T.H. (1990). Protein Splicing Converts the Yeast TFP1 Gene Product to the 69-kDa Subunit of the Vacuolar H^+^-Adenosine Triphosphatase. Science.

[10] Hirata R., Ohsumi Y., Nakano A., Kawasaki H., Suzuki K., Anraku Y. (1990). Molecular Structure of a Gene, *VMA1*, Encoding the Catalytic Subunit of H^+^-Translocating Adenosine Triphosphatase from Vacuolar Membranes of *Saccharomyces cerevisiae*. J. Biol. Chem..

[11] Perler F.B. (2002). InBase: The Intein Database. Nucleic Acids Res..

[12] Mills K.V., Johnson M.A., Perler F.B. (2014). Protein Splicing: How Inteins Escape from Precursor Proteins. J. Biol. Chem..

[13] Prabhala S.V., Gierach I., Wood D.W. (2022). The Evolution of Intein-Based Affinity Methods as Reflected in 30 Years of Patent History. Front. Mol. Biosci..

[14] Gogarten J.P., Hilario E. (2006). Inteins, Introns, and Homing Endonucleases: Recent Revelations about the Life Cycle of Parasitic Genetic Elements. BMC Evol. Biol..

[15] Novikova O., Topilina N., Belfort M. (2014). Enigmatic Distribution, Evolution, and Function of Inteins. J. Biol. Chem..

[16] Shah N.H., Muir T.W. (2014). Inteins: Nature’s Gift to Protein Chemists. Chem. Sci..

[17] Hirata R., Anraku Y. (1992). Mutations at the Putative Junction Sites of the Yeast VMA1 Protein, the Catalytic Subunit of the Vacuolar Membrane H^+^-ATPase, Inhibit Its Processing by Protein Splicing. Biochem. Biophys. Res. Commun..

[18] Wu H., Hu Z., Liu X.-Q. (1998). Protein Trans-Splicing by a Split Intein Encoded in a Split DnaE Gene of *Synechocystis* sp. PCC6803. Proc. Natl. Acad. Sci. USA.

[19] Evans T.C., Martin D., Kolly R., Panne D., Sun L., Ghosh I., Chen L., Benner J., Liu X.-Q., Xu M.-Q. (2000). Protein Trans-Splicing and Cyclization by a Naturally Split Intein from the *dnaE* Gene of *Synechocystis* Species PCC6803. J. Biol. Chem..

[20] Brenzel S., Kurpiers T., Mootz H.D. (2006). Engineering Artificially Split Inteins for Applications in Protein Chemistry: Biochemical Characterization of the Split Ssp DnaB Intein and Comparison to the Split Sce VMA Intein. Biochemistry.

[21] Lin Y., Li M., Song H., Xu L., Meng Q., Liu X.-Q. (2013). Protein Trans-Splicing of Multiple Atypical Split Inteins Engineered from Natural Inteins. PLoS ONE.

[22] Song H., Meng Q., Liu X.-Q. (2012). Protein Trans-Splicing of an Atypical Split Intein Showing Structural Flexibility and Cross-Reactivity. PLoS ONE.

[23] Wood D.W., Belfort M., Lennon C.W. (2023). Inteins—Mechanism of Protein Splicing, Emerging Regulatory Roles, and Applications in Protein Engineering. Front. Microbiol..

[24] Anastassov S., Filo M., Khammash M. (2024). Inteins: A Swiss Army Knife for Synthetic Biology. Biotechnol. Adv..

[25] Thiel I.V., Volkmann G., Pietrokovski S., Mootz H.D. (2014). An Atypical Naturally Split Intein Engineered for Highly Efficient Protein Labeling. Angew. Chem. Int. Ed. Engl..

[26] Li X., Zhang L., Wang S., Liu X., Lin Y. (2021). Site-Specific Internal Protein Labeling through Trans-Splicing. Int. J. Biol. Macromol..

[27] Ewen-Campen B., Luan H., Xu J., Singh R., Joshi N., Thakkar T., Berger B., White B.H., Perrimon N. (2023). Split-Intein Gal4 Provides Intersectional Genetic Labeling That Is Repressible by Gal80. Proc. Natl. Acad. Sci. USA.

[28] Cooper M.A., Taris J.E., Shi C., Wood D.W. (2018). A Convenient Split-Intein Tag Method for the Purification of Tagless Target Proteins. Curr. Protoc. Protein Sci..

[29] Zhao Q., Xu W., Xing L., Lin Z. (2016). Recombinant Production of Medium-to-Large-Sized Peptides in *Escherichia coli* Using a Cleavable Self-Aggregating Tag. Microb. Cell Fact..

[30] Ferreira M.V., Fernandes S., Almeida A.I., Neto S., Mendes J.P., Silva R.J.S., Peixoto C., Coroadinha A.S. (2023). Extending AAV Packaging Cargo through Dual Co-Transduction: Efficient Protein Trans-Splicing at Low Vector Doses. Int. J. Mol. Sci..

[31] Schaerli Y., Gili M., Isalan M. (2014). A Split Intein T7 RNA Polymerase for Transcriptional AND-Logic. Nucleic Acids Res..

[32] Olorunniji F.J., Lawson-Williams M., McPherson A.L., Paget J.E., Stark W.M., Rosser S.J. (2019). Control of ϕC31 Integrase-Mediated Site-Specific Recombination by Protein Trans-Splicing. Nucleic Acids Res..

[33] Wright J.N., Wong W.L., Harvey J.A., Garnett J.A., Itzhaki L.S., Main E.R.G. (2019). Scalable Geometrically Designed Protein Cages Assembled via Genetically Encoded Split Inteins. Structure.

[34] Qi X., Wang J., Meng Q., Liu X.-Q. (2011). Alternative Nucleophilic Residues in Intein Catalysis of Protein Splicing. Protein Pept. Lett..

[35] Bachmann A.L., Mootz H.D. (2015). An Unprecedented Combination of Serine and Cysteine Nucleophiles in a Split Intein with an Atypical Split Site. J. Biol. Chem..

[36] Aranko A.S., Wlodawer A., Iwai H. (2014). Nature’s Recipe for Splitting Inteins. Protein Eng. Des. Sel..

[37] Xu M.Q., Perler F.B. (1996). The Mechanism of Protein Splicing and Its Modulation by Mutation. EMBO J..

[38] Tori K., Dassa B., Johnson M.A., Southworth M.W., Brace L.E., Ishino Y., Pietrokovski S., Perler F.B. (2010). Splicing of the Mycobacteriophage Bethlehem DnaB Intein: Identification of a New Mechanistic Class of Inteins That Contain an Obligate Block F Nucleophile. J. Biol. Chem..

[39] Shah N.H., Eryilmaz E., Cowburn D., Muir T.W. (2013). Naturally Split Inteins Assemble through a “Capture and Collapse” Mechanism. J. Am. Chem. Soc..

[40] Bhagawati M., Hoffmann S., Höffgen K.S., Piehler J., Busch K.B., Mootz H.D. (2020). In Cellulo Protein Semi-Synthesis from Endogenous and Exogenous Fragments Using the Ultra-Fast Split Gp41-1 Intein. Angew. Chem. Int. Ed. Engl..

[41] Gordo V., Aparicio D., Pérez-Luque R., Benito A., Vilanova M., Usón I., Fita I., Ribó M. (2018). Structural Insights into Subunits Assembly and the Oxyester Splicing Mechanism of Neq Pol Split Intein. Cell Chem. Biol..

[42] Hoffmann S., Terhorst T.M.E., Singh R.K., Kümmel D., Pietrokovski S., Mootz H.D. (2021). Biochemical and Structural Characterization of an Unusual and Naturally Split Class 3 Intein. Chembiochem.

[43] Stevens A.J., Sekar G., Gramespacher J.A., Cowburn D., Muir T.W. (2018). An Atypical Mechanism of Split Intein Molecular Recognition and Folding. J. Am. Chem. Soc..

[44] Ciragan A., Aranko A.S., Tascon I., Iwaï H. (2016). Salt-Inducible Protein Splicing in *cis* and *trans* by Inteins from Extremely Halophilic Archaea as a Novel Protein-Engineering Tool. J. Mol. Biol..

[45] Heikkinen H.A., Aranko A.S., Iwaï H. (2022). The NMR Structure of the Engineered Halophilic DnaE Intein for Segmental Isotopic Labeling Using Conditional Protein Splicing. J. Magn. Reson..

[46] Callahan B.P., Topilina N.I., Stanger M.J., Van Roey P., Belfort M. (2011). Structure of Catalytically Competent Intein Caught in a Redox Trap with Functional and Evolutionary Implications. Nat. Struct. Mol. Biol..

[47] Berrade L., Kwon Y., Camarero J.A. (2010). Photomodulation of Protein Trans-Splicing through Backbone Photocaging of the DnaE Split Intein. Chembiochem.

[48] Ren W., Ji A., Ai H.W. (2015). Light Activation of Protein Splicing with a Photocaged Fast Intein. J. Am. Chem. Soc..

[49] Vila-Perelló M., Hori Y., Ribó M., Muir T.W. (2008). Activation of Protein Splicing by Protease- or Light-Triggered O to N Acyl Migration. Angew. Chem. Int. Ed. Engl..

[50] Swartz T.E., Corchnoy S.B., Christie J.M., Lewis J.W., Szundi I., Briggs W.R., Bogomolni R.A. (2001). The Photocycle of a Flavin-Binding Domain of the Blue Light Photoreceptor Phototropin. J. Biol. Chem..

[51] Harper S.M., Neil L.C., Gardner K.H. (2003). Structural Basis of a Phototropin Light Switch. Science.

[52] Losi A., Gardner K.H., Möglich A. (2018). Blue-Light Receptors for Optogenetics. Chem. Rev..

[53] Wong S., Mosabbir A.A., Truong K. (2015). An Engineered Split Intein for Photoactivated Protein Trans-Splicing. PLoS ONE.

[54] Gramespacher J.A., Stevens A.J., Nguyen D.P., Chin J.W., Muir T.W. (2017). Intein Zymogens: Conditional Assembly and Splicing of Split Inteins via Targeted Proteolysis. J. Am. Chem. Soc..

[55] Zeidler M.P., Tan C., Bellaiche Y., Cherry S., Häder S., Gayko U., Perrimon N. (2004). Temperature-Sensitive Control of Protein Activity by Conditionally Splicing Inteins. Nat. Biotechnol..

[56] Mootz H.D., Muir T.W. (2002). Protein Splicing Triggered by a Small Molecule. J. Am. Chem. Soc..

[57] Inobe T., Nukina N. (2016). Rapamycin-Induced Oligomer Formation System of FRB-FKBP Fusion Proteins. J. Biosci. Bioeng..

[58] Mootz H.D., Blum E.S., Tyszkiewicz A.B., Muir T.W. (2003). Conditional Protein Splicing: A New Tool to Control Protein Structure and Function in Vitro and in Vivo. J. Am. Chem. Soc..

[59] Sonntag T., Mootz H.D. (2011). An Intein-Cassette Integration Approach Used for the Generation of a Split TEV Protease Activated by Conditional Protein Splicing. Mol. Biosyst..

[60] Gramespacher J.A., Burton A.J., Guerra L.F., Muir T.W. (2019). Proximity Induced Splicing Utilizing Caged Split Inteins. J. Am. Chem. Soc..

[61] Lonzarić J., Lebar T., Majerle A., Manček-Keber M., Jerala R. (2016). Locked and Proteolysis-Based Transcription Activator-Like Effector (TALE) Regulation. Nucleic Acids Res..

[62] Zettler J., Schütz V., Mootz H.D. (2009). The Naturally Split Npu DnaE Intein Exhibits an Extraordinarily High Rate in the Protein Trans-Splicing Reaction. FEBS Lett..

[63] Iwai H., Zuger S., Jin J., Tam P.H. (2006). Highly Efficient Protein Trans-Splicing by a Naturally Split DnaE Intein from *Nostoc punctiforme*. FEBS Lett..

[64] Stevens A.J., Brown Z.Z., Shah N.H., Sekar G., Cowburn D., Muir T.W. (2016). Design of a Split Intein with Exceptional Protein Splicing Activity. J. Am. Chem. Soc..

[65] Beyer H.M., Mikula K.M., Li M., Wlodawer A., Iwai H. (2020). The Crystal Structure of the Naturally Split gp41-1 Intein Guides the Engineering of Orthogonal Split Inteins from Cis-Splicing Inteins. FEBS J..

[66] Martin D.D., Xu M.Q., Evans T.C. (2001). Characterization of a Naturally Occurring Trans-Splicing Intein from *Synechocystis* sp. PCC6803. Biochemistry.

[67] Appleby J.H., Zhou K., Volkmann G., Liu X.Q. (2009). Novel Split Intein for Trans-Splicing Synthetic Peptide onto C Terminus of Protein. J. Biol. Chem..

[68] Sun W., Yang J., Liu X.Q. (2004). Synthetic Two-Piece and Three-Piece Split Inteins for Protein Trans-Splicing. J. Biol. Chem..

[69] Volkmann G., Liu X.Q. (2011). Intein Lacking Conserved C-Terminal Motif G Retains Controllable N-Cleavage Activity. FEBS J..

[70] Li X., Zhang X.L., Cai Y.M., Zhang L., Lin Y., Meng Q. (2018). Site-Specific Labeling of Two Proteins in One System by Atypical Split Inteins. Int. J. Biol. Macromol..

[71] Shi J., Muir T.W. (2005). Development of a Tandem Protein Trans-Splicing System Based on Native and Engineered Split Inteins. J. Am. Chem. Soc..

[72] Matern J.C., Bachmann A.L., Thiel V.I., Volkmann G., Wasmuth A., Binschik J., Mootz H.D. (2015). Ligation of Synthetic Peptides to Proteins Using Semisynthetic Protein Trans-Splicing. Methods Mol. Biol..

[73] Truong D.J., Kühner K., Kühn R., Werfel S., Engelhardt S., Wurst W., Ortiz O. (2015). Development of an Intein-Mediated Split-Cas9 System for Gene Therapy. Nucleic Acids Res..

[74] Cheriyan M., Pedamallu C.S., Tori K., Perler F. (2013). Faster Protein Splicing with the *Nostoc punctiforme* DnaE Intein Using Non-Native Extein Residues. J. Biol. Chem..

[75] Shah N.H., Eryilmaz E., Cowburn D., Muir T.W. (2013). Extein Residues Play an Intimate Role in the Rate-Limiting Step of Protein Trans-Splicing. J. Am. Chem. Soc..

[76] Stevens A.J., Sekar G., Shah N.H., Mostafavi A.Z., Cowburn D., Muir T.W. (2017). A Promiscuous Split Intein with Expanded Protein Engineering Applications. Proc. Natl. Acad. Sci. USA.

[77] Aranko A.S., Oeemig J.S., Zhou D., Kajander T., Wlodawer A., Iwaï H. (2014). Structure-Based Engineering and Comparison of Novel Split Inteins for Protein Ligation. Mol. Biosyst..

[78] Beauchamp D.L., Khajehpour M. (2012). Studying Salt Effects on Protein Stability Using Ribonuclease T1 as a Model System. Biophys. Chem..

[79] Xia H.F., Luo J.P., Yu S.R., Zhou T.J. (2022). Modification of C-Segment of Cfa DnaE Split Intein for Improving Clean-in-Place in Chromatography Process. Biotechnol. Prog..

[80] Ludwig C., Schwarzer D., Mootz H.D. (2008). Interaction Studies and Alanine Scanning Analysis of a Semi-Synthetic Split Intein Reveal Thiazoline Ring Formation from an Intermediate of the Protein Splicing Reaction. J. Biol. Chem..

[81] Zhang X., Liu X.Q., Meng Q. (2019). Engineered Ssp DnaX Inteins for Protein Splicing with Flanking Proline Residues. Saudi J. Biol. Sci..

[82] Kurpiers T., Mootz H.D. (2008). Site-Specific Chemical Modification of Proteins with a Prelabelled Cysteine Tag Using the Artificially Split Mxe GyrA Intein. Chembiochem.

[83] Lee E., Choi H.K., Kwon Y., Lee K.B. (2024). Real-Time, Non-Invasive Monitoring of Neuronal Differentiation Using Intein-Enabled Fluorescence Signal Translocation in Genetically Encoded Stem Cell-Based Biosensors. Adv. Funct. Mater..

[84] Prabhala S.V., Marshall B., Galiardi J., Fan Y., Creamer E., Wood D.W. (2024). Highly selective split intein method for efficient separation and purification of recombinant therapeutic proteins from mammalian cell culture fluid. J. Chromatogr. A.

[85] Sevillano E., Lafuente I., Peña N., Cintas L.M., Muñoz-Atienza E., Hernández P.E., Borrero J. (2024). Isolation, Genomics-Based and Biochemical Characterization of Bacteriocinogenic Bacteria and Their Bacteriocins, Sourced from the Gastrointestinal Tract of Meat-Producing Pigs. Int. J. Mol. Sci..

[86] McDermott A., Windeln L.M., Valentine J.S.D., Baldassarre L., Foster A.D., Tavassoli A. (2024). Next Generation SICLOPPS Screening for the Identification of Inhibitors of the HIF-1α/HIF-1β Protein-Protein Interaction. ACS Chem. Biol..

[87] Jumper J., Evans R., Pritzel A., Green T., Figurnov M., Ronneberger O., Tunyasuvunakool K., Bates R., Žídek A., Potapenko A. (2021). Highly Accurate Protein Structure Prediction with AlphaFold. Nature.

[88] Evans R., O’Neill M., Pritzel A., Antropova N., Senior A., Green T., Žídek A., Bates R., Blackwell S., Yim J. Protein Complex Prediction with AlphaFold-Multimer. bioRxiv.

[89] Abramson J., Adler J., Dunger J., Evans R., Green T., Pritzel A., Ronneberger O., Willmore L., Ballard A.J., Bambrick J. (2024). Accurate Structure Prediction of Biomolecular Interactions with AlphaFold 3. Nature.

